# Laparoscopic Cholecystectomy for Necrotic Perforated Cholecystitis in a Super-obese, Highly Frail Patient: A Case Report

**DOI:** 10.7759/cureus.101869

**Published:** 2026-01-19

**Authors:** Miray Yilmaz, Angel Oluwatobi Okpe, Asal Sabouri, Belis Sude Kemanci, Servet Karagul

**Affiliations:** 1 General Surgery, Faculty of Medicine, Istanbul Atlas University, Istanbul, TUR

**Keywords:** cholecystitis, frailty, minimal invasive, morbid obesity, perforation of gallbladder

## Abstract

Acute necrotic perforated cholecystitis is a life-threatening condition requiring urgent surgical treatment. The risk of morbidity and mortality increases in patients with high frailty. Here, we present a rare case of necrotic perforated cholecystitis treated with laparoscopic cholecystectomy in a 72-year-old female patient with a BMI of 54.6 kg/m^2^, diabetes, a recent cerebrovascular event, and cardiac and pulmonary diseases. She presented with abdominal pain after being hospitalized for an acute ischemic stroke two weeks prior. CT scan revealed gangrenous cholecystitis and gallbladder perforation. The patient underwent a laparoscopic cholecystectomy. No complications were observed in the postoperative period. The patient was discharged without complications. Laparoscopic cholecystectomy can be safely applied in selected patients with necrotic perforated cholecystitis who are super obese and have very high frailty due to comorbidities.

## Introduction

Acute necrotic perforated cholecystitis requires urgent surgical intervention. Gallbladder perforation occurs in 2-15% of patients with acute cholecystitis and is associated with a mortality rate of up to 7% [1]. It is particularly prevalent among the elderly and individuals with comorbidities. Gangrenous cholecystitis is a more severe form of the disease, which progresses rapidly and is associated with high morbidity and mortality rates [2]. Morbid obesity and diabetes mellitus (DM) can lead to delays in diagnosis and pose technical challenges, particularly in cases involving gangrenous or perforated gallbladders. Perioperative complications are also more common [3,4].

Open cholecystectomy is conventionally the preferred method for complicated gallbladders, particularly in patients at very high risk. However, laparoscopic techniques are increasingly being used for some at-risk patients. When performed by experienced surgeons, laparoscopic cholecystectomy can be beneficial for perforated gallbladders, resulting in reduced postoperative pain, a shorter hospital stay and a faster recovery [5,6]. When meticulous surgical preparation is performed, minimally invasive surgery can be suitable for high-risk patients.

Here, we present the case of a 72-year-old diabetic patient with gangrenous, necrotic and perforated cholecystitis who had recently suffered a heart attack and cerebrovascular embolism. Despite her complicated medical history, we performed an emergency laparoscopic cholecystectomy. This case demonstrates that laparoscopic surgery can be beneficial for high-risk patients with necrotic, perforated cholecystitis, including morbidly obese diabetic patients.

## Case presentation

A 72-year-old female patient with a body mass index (BMI) of 54.6 kg/m^2^ was admitted to the neurology department two weeks earlier with a history of acute ischemic stroke. She had been experiencing abdominal pain for one week. When the pain gradually worsened and was later accompanied by vomiting and nausea, the general surgery team was consulted.

**Figure 1:**
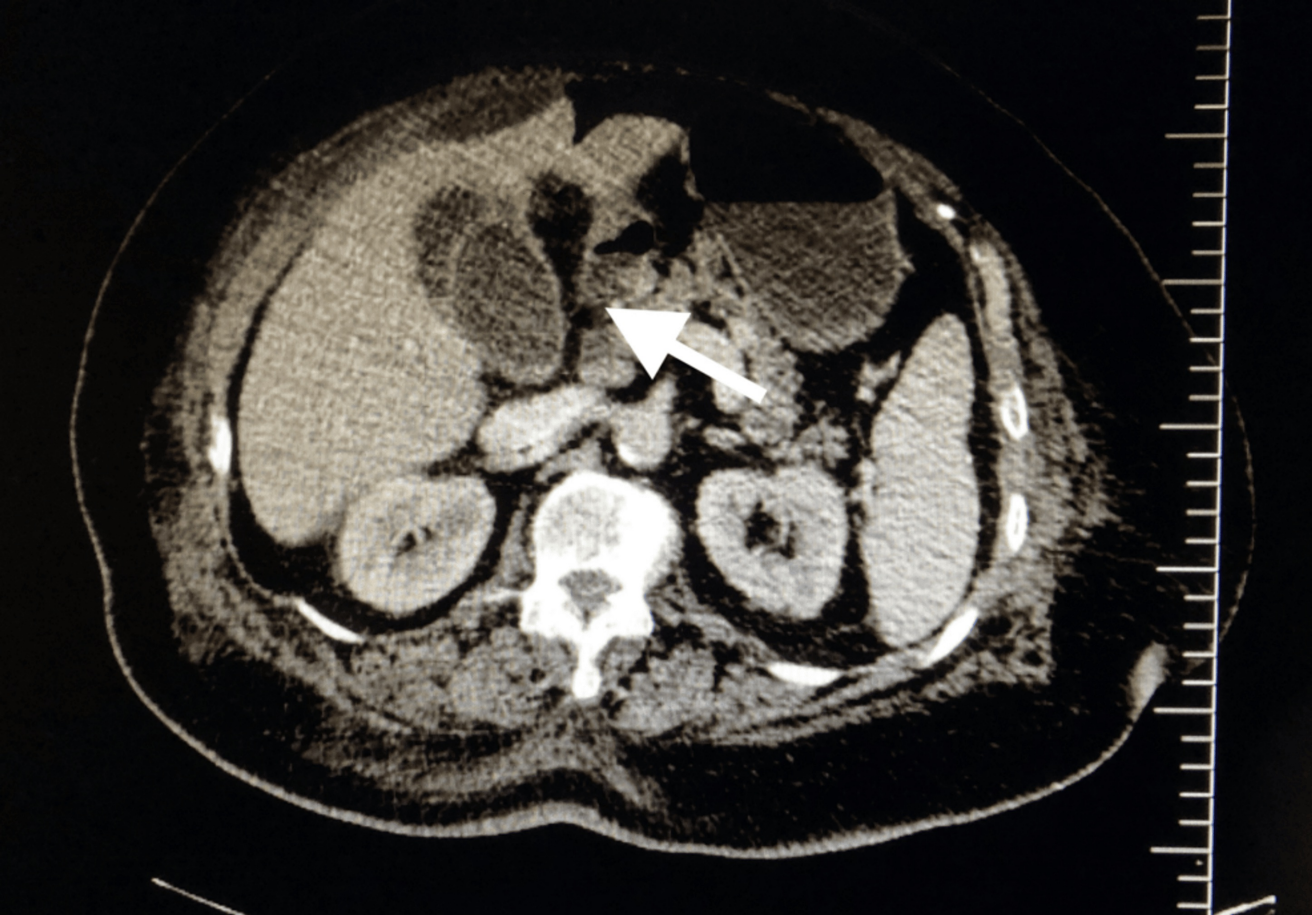
CT image of the patient; the arrow indicates a perforated gallbladder.

The patient's medical history includes a myocardial infarction (MI) six months ago and an ischemic stroke two weeks ago. She has been diagnosed with DM, hypertension, and chronic obstructive pulmonary disease (COPD). She is currently taking aspirin, warfarin, and insulin, in addition to cardiovascular agents. On physical examination, her general condition was assessed as moderate: she appeared to be both morbidly obese and paraplegic. Abdominal examination revealed diffuse tenderness and guarding. Laboratory findings revealed a white blood cell count of 13,300 μL, a C-reactive protein level of 36 mg/dL and an international normalised ratio (INR) of 6.37. Radiological imaging, including ultrasound and computed tomography, revealed findings consistent with gangrenous cholecystitis and gallbladder perforation (Figure 1).

**Figure 2:**
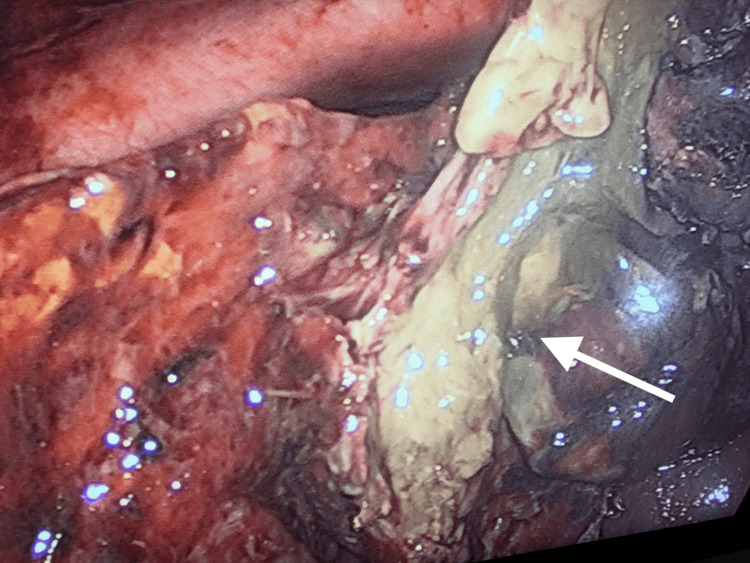
Laparoscopic view of the surgical field; the arrow indicates the necrotic gallbladder.

**Figure 3:**
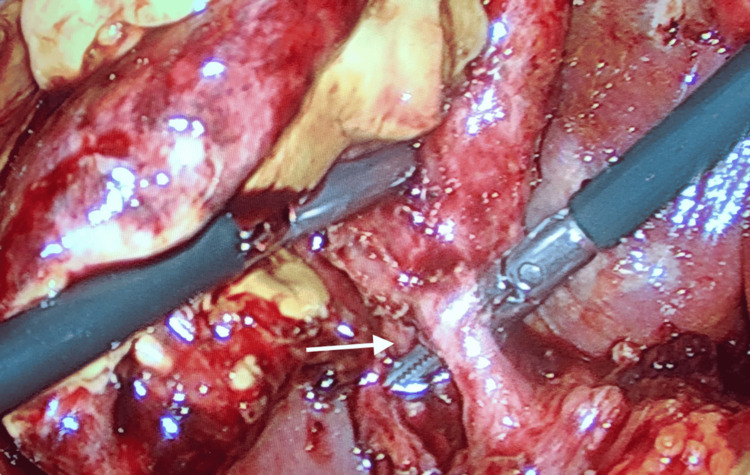
Intraoperative laparoscopic view demonstrating safe surgical anatomy; the arrow indicates the cystic duct.

**Figure 4:**
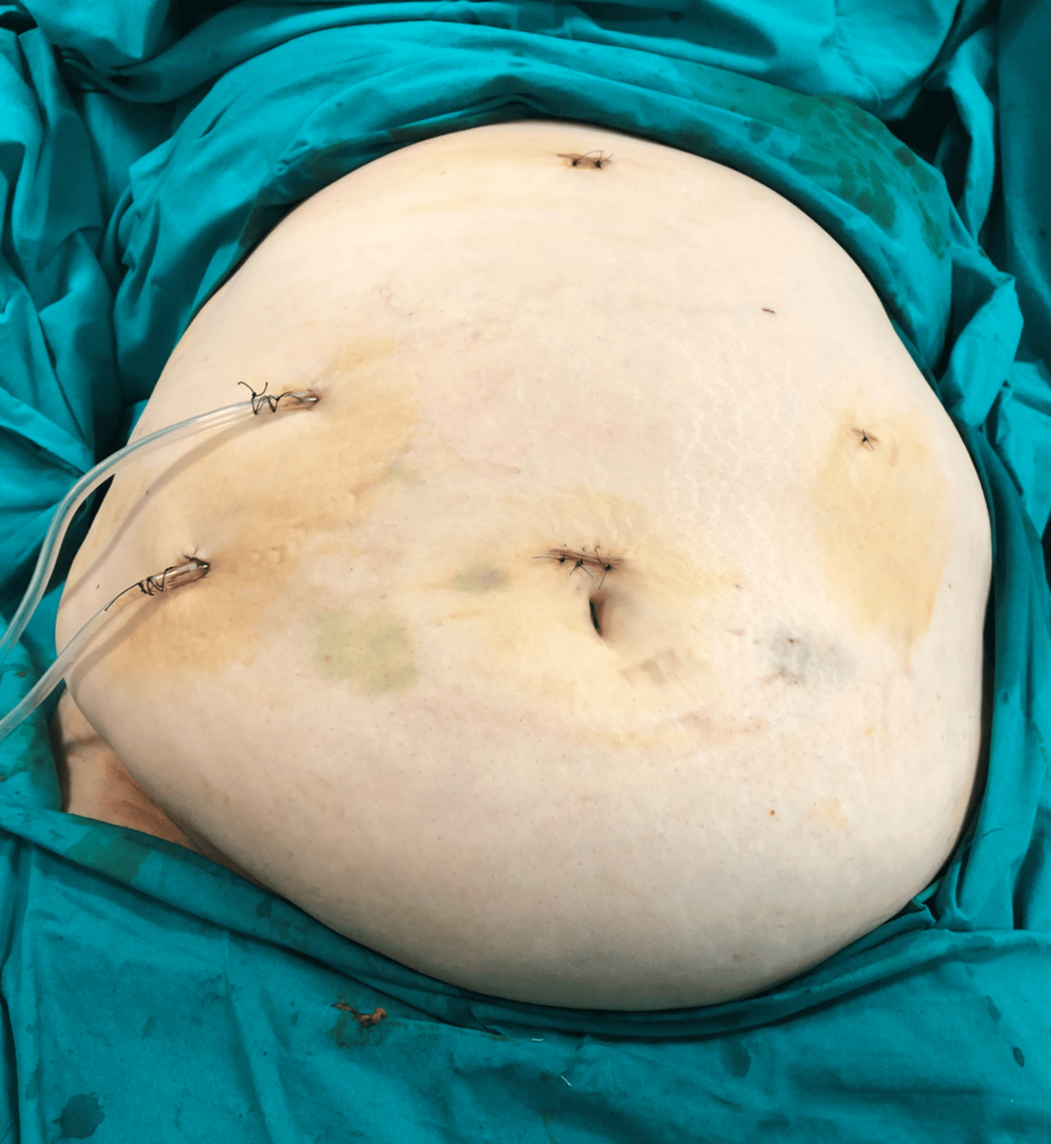
Postoperative view of the abdomen following the laparoscopic procedure.

Oral intake was withheld, and intravenous fluid therapy was commenced. Oral anticoagulant medication was stopped. The patient received fresh frozen plasma transfusions, and her INR was rechecked and found to be 2.8 before the operation. Intravenous antibiotherapy with ceftriaxone and metronidazole was initiated. The patient underwent an emergency laparoscopic cholecystectomy eight hours later. Exploration revealed severe adhesions and abscesses in the upper right quadrant of the abdomen. After aspirating the abscessed collections, a necrotic, perforated gallbladder was observed (Figure 2). Once the cystic duct and artery were clearly identified, the laparoscopic procedure was maintained (Figure 3). Both structures were ligated separately with clips and cut. The gallbladder was separated from the liver bed using sharp dissection and removed through the trocar site in a specimen bag. One drain was placed in the foramen of Winslow and another in the right subdiaphragmatic space (see Figure 4). On postoperative day 1, her oral intake was initiated, and her abdominal examination revealed no abnormalities. On postoperative day 3, however, she developed pneumonia and received appropriate treatment. While the patient was clinically stable on postoperative day 7, warfarin treatment was started via bridging therapy. She was discharged on postoperative day 9. No problems were encountered during follow-up.

## Discussion

The treatment of a necrotic gallbladder and perforation of the gallbladder in a morbidly obese and diabetic patient poses a complex surgical challenge, given the associated perioperative risks and postoperative complications. Adding to the complexity of this case, the patient has a history of ischaemic stroke two weeks prior and MI six months prior. Furthermore, with a BMI of 54.6 kg/m^2^, the patient is categorised as super-obese. Conversion rate is significantly associated with acute cholecystitis, obesity and emergency surgery in laparoscopic cholecystectomy [7]. In the past, morbid obesity was considered a relative contraindication for laparoscopic cholecystectomy. Current evidence has shown that laparoscopic cholecystectomy can be performed safely in the morbid and super obese patients and with the same favourable outcomes as seen in patients with a normal BMI [8]. Inflammation, adhesions, anatomical difficulties and increased visceral fat in obese patients increase the risk of reduced visibility and manoeuvrability, thus increasing the risk of conversion to open surgery [9]. However, considering that in a patient with perforated necrotic cholecystitis like the one we presented, open surgery would increase surgical site infection and be very difficult to manage, we found it appropriate to start the operation with laparoscopic surgery. A meta-analysis in the literature reported that laparoscopic surgery in obese patients reduces the surgical site infection rate by 70-80% compared with open surgery across general abdominal surgical procedures, and we also preferred laparoscopy in our case due to the high need for this advantage [10].

Gallbladder perforation is a rare and serious condition requiring emergency intervention. Surgical intervention and drainage, antibiotic therapy, and fluid resuscitation are critically important. A significant proportion of patients diagnosed with this condition have been found to be obese [11]. Obesity can increase the risk of gallbladder perforation through its accompanying comorbidities [5,11]. In particular, DM can lead to the development of neuropathy, masking of classic symptoms until perforation occurs and delay in diagnosis. In addition, diabetes can impair the function of white blood cells, leading to immunosuppression in the patient [11]. Aydoğdu et al. reported the presence of DM in 42.9% of gallbladder perforation cases [6]. In our case, although a serious necrosis and perforation developed, the severity of the symptoms was not as expected, and only when nausea and vomiting occurred were the clinical findings taken seriously, and the patient was consulted in the neurology ward, where he was admitted. Our patient was also at risk from a cardiac perspective and had a history of MI six months prior. It is stated that the incidence of acute cholecystitis is higher in acute stroke patients. Acute cholecystitis should be suspected after each general disease leading to hypoperfusion, such as cardiovascular diseases or cerebrovascular diseases [12,13]. Regardless of aetiology, visceral hypoperfusion, ischemia and reperfusion injury, and bile stasis play a role in the pathogenesis of acute cholecystitis in these patients.

Fagenson et al.'s cohort analysis, which aimed to determine the association between frailty and postoperative morbidity and mortality in patients undergoing laparoscopic cholecystectomy for acute cholecystitis, is a valuable study for identifying high-risk patients. According to this study, individual frailty components associated with Clavien IV complications, such as DM, a history of MI within six months, cerebrovascular accident, hypertension and COPD, were present in our case [14]. Furthermore, the same study identified being over 65 years of age and having a BMI above 30 as factors associated with mortality and Clavien IV complications; these risks were also present in our case. We believed that performing open surgery on such a patient would make it difficult to avoid factors that would increase morbidity and mortality, primarily postoperative pulmonary problems and surgical site infections. Despite these challenges, laparoscopic cholecystectomy enabled us to successfully treat a patient with high frailty, necrotic and perforated acute cholecystitis.

Minimally invasive surgery is favoured for elective procedures due to faster recovery times, reduced pain, shorter hospital stays and a lower risk of wound complications. This is particularly beneficial in surgeries on morbidly obese patients, as it minimises problems arising from the surgical site. We can prefer the minimally invasive method in patients with diabetes, cardiovascular diseases, acute cerebrovascular problems, chronic pulmonary disease and necrotic and perforated cholecystitis. Serious complications related to the surgical incision sites will occur in a high-risk patient with these conditions, and treatment will also be arduous. A laparoscopic cholecystectomy can be safely performed on highly frail, super-obese patients with necrotic, perforated acute cholecystitis.

## Conclusions

Minimally invasive surgery is favoured for elective procedures due to faster recovery times, reduced pain, shorter hospital stays and a lower risk of wound complications. This is particularly beneficial in surgeries on morbidly obese patients, as it minimises problems arising from the surgical site. We can prefer the minimally invasive method in patients with diabetes, cardiovascular diseases, acute cerebrovascular problems, chronic pulmonary disease and necrotic and perforated cholecystitis. Serious complications related to the surgical incision sites will occur in a high-risk patient with these conditions, and treatment will also be arduous. A laparoscopic cholecystectomy can be safely performed on highly frail, super-obese patients with necrotic, perforated acute cholecystitis.
